# Hunting Down Frame Shifts: Ecological Analysis of Diverse Functional Gene Sequences

**DOI:** 10.3389/fmicb.2015.01267

**Published:** 2015-11-24

**Authors:** Michal Strejcek, Qiong Wang, Jakub Ridl, Ondrej Uhlik

**Affiliations:** ^1^Department of Biochemistry and Microbiology, Faculty of Food and Biochemical Technology, University of Chemistry and Technology, PraguePrague, Czech Republic; ^2^Center for Microbial Ecology, Michigan State UniversityEast Lansing, MI, USA; ^3^Department of Genomics and Bioinformatics, Institute of Molecular Genetics, Academy of Sciences of the Czech RepublicPrague, Czech Republic

**Keywords:** Frameshift, FrameBot, biphenyl dioxygenase, benzoate dioxygenase, amplicon sequencing, functional genes

## Abstract

Functional gene ecological analyses using amplicon sequencing can be challenging as translated sequences are often burdened with shifted reading frames. The aim of this work was to evaluate several bioinformatics tools designed to correct errors which arise during sequencing in an effort to reduce the number of frameshifts (FS). Genes encoding for alpha subunits of biphenyl (*bphA*) and benzoate (*benA*) dioxygenases were used as model sequences. FrameBot, a FS correction tool, was able to reduce the number of detected FS to zero. However, up to 44% of sequences were discarded by FrameBot as non-specific targets. Therefore, we proposed a *de novo* mode of FrameBot for FS correction, which works on a similar basis as common chimera identifying platforms and is not dependent on reference sequences. By nature of FrameBot *de novo* design, it is crucial to provide it with data as error free as possible. We tested the ability of several publicly available correction tools to decrease the number of errors in the data sets. The combination of maximum expected error filtering and single linkage pre-clustering proved to be the most efficient read processing approach. Applying FrameBot *de novo* on the processed data enabled analysis of BphA sequences with minimal losses of potentially functional sequences not homologous to those previously known. This experiment also demonstrated the extensive diversity of dioxygenases in soil. A script which performs FrameBot *de novo* is presented in the supplementary material to the study or available at https://github.com/strejcem/FBdenovo. The tool was also implemented into FunGene Pipeline available at http://fungene.cme.msu.edu/FunGenePipeline/.

## Introduction

Next generation sequencing (NGS) of amplicons has become a standard method for assessing diversity in microbial ecology. In particular, the 16S rRNA gene as a taxonomic marker is heavily sequenced and many software pipelines have been developed for its processing, such as mothur (Schloss et al., 2009), Ribosomal Database Project (RDP) pipeline (Cole et al., 2014), QIIME (Caporaso et al., 2010), and others. The general workflow for processing of amplicon sequence data is to first reduce sequencing errors (Schloss et al., 2011), then eliminate chimeric reads (Edgar et al., 2011; Quince et al., 2011), and finally form operational taxonomic units (OTUs). Resultant OTUs are classified and analyzed, i.e., alpha and beta diversity measurements are carried out, and statistical descriptions/analyses are performed (Schloss et al., 2011). Such strategies are very efficient in masking PCR-generated and sequencing errors and do not radically inflate diversity estimates, which was historically a major problem of NGS of amplicons (Quince et al., 2009; Huse et al., 2010).

When interested in investigating specific ecological processes, functional diversity is often more informative than taxonomic diversity as it asks *which* functional genes are present rather than *who* is present. One major potential problem associated with analysis of amplicon sequenced functional genes is the possibility of shifted reading frames. Universal strategies which exist for 16S rRNA gene processing do not yet exist for functional genes, although both RDP’s FunGene Pipeline (Fish et al., 2013) and FunFrame (Weisman et al., 2013) represent certain standardizations of the analyses. Both pipelines correct frameshifts (FS) through different algorithms; FunGene employs FrameBot (Wang et al., 2013), whereas FunFrame uses HMM-FRAME (Zhang and Sun, 2011). Wang et al. (2013) reported that FrameBot outperformed HMM-FRAME in terms of FS correction. Both pipelines also reported that FS in very diverse sets of environmental sequences could not be efficiently eliminated, which stems from incomplete databases not reflecting actual diversity. An example of such a problematic case is the analysis of biphenyl dioxygenase (*bphA*) gene. BphA is the alpha subunit of biphenyl dioxygenase and its gene is traditionally considered as the genetic marker for biphenyl and polychlorinated biphenyls (PCBs) utilization. The upper biphenyl degradation pathway (Furukawa et al., 1989) results in the production of hydroxypentadienoate and benzoate, which then enter the lower degradation pathway. The lower pathway for the catabolism of (chloro)benzoates is initiated by another dioxygenase, benzoate dioxygenase, encoded by the *benA* gene (Pieper and Seeger, 2008).

In this study, we tested three different methods of processing amplicon sequence data of functional genes using specific example data sets of biphenyl and benzoate dioxygenases. Dioxygenases were picked intentionally due to their extensive diversity and low coverage of known sequences which can be used as references (Wang et al., 2013). More specifically, bioinformatics approaches using denoising or maximum expected error (MEE) trimming were compared, and a novel stand-alone method of FS corrections, FrameBot *de novo*, is proposed which enables analyses independent of previously described sequences.

## Materials and Methods

### Soil and Mock Community Samples

Two different soils were used for DNA isolation in this study. The first was a long-term contaminated soil from a dump site in south Bohemia mainly polluted with PCBs originating from Delor 103 and Delor 106 mixtures (Pavlíková et al., 2007), as well as polyaromatic hydrocarbons, pesticides, heavy metals, and other pollutants (Uhlík et al., 2012). The second soil was a pristine soil used commonly as horticultural substrate. In addition, a collection of bacterial genomes carrying the *bphA* and *benA* genes was prepared as a mock community from four strains: *Burkholderia xenovorans* LB400 (Bopp, 1986), *Rhodococcus jostii* RHA1 (Masai et al., 1995), *Pseudomonas alcaliphila* JAB1 (Ryšlavá et al., 2003; Kurzawová et al., 2012), *Pandoraea pnomenusa* (formerly *Comamonas testosteroni*) B-356 (Hurtubise et al., 1995).

### DNA Isolation and Amplicon Preparation

Total DNA was extracted from both soils with PowerMax^TM^ Soil DNA Isolation Kit (MoBio Laboratories Inc., USA) following the standard protocol. Ethanol precipitation with glycogen (Roche, Germany) was carried out to further concentrate the DNA as was described by Uhlík et al. (2009). DNA of mock community strains was isolated using PureLink Genomic DNA Mini Kit (Invitrogen, USA) after the strains were grown overnight in liquid Luria–Bertani medium (Oxoid, UK). Prior to amplification, genomic DNA of the strains was pooled and further processed analogously to soil metagenomic DNA.

Amplicons were prepared by PCR with primers fused with unique barcode sequences enabling to distinguish individual samples. Primers for *bphA* were adapted from Iwai et al. (2010). A new set of degenerated primers was designed for *benA* based on known sequences available in RDP FunGene database (Fish et al., 2013). Known sequences of *benA* which were >400 bp in size and >900 in score were downloaded and aligned, and primers were designed in the conserved regions of the gene. The novel set of primers is as follows: *benA* 649f, GAR AAY GGH GCN GAY GGY TAY CA; and *benA* 1100r, AAR AAR TCY TCR TAY TGR CG (numbering based on *B. xenovorans* LB400 *benA*). The primers were tested with genomic DNA of the strains contained in the mock community. PCR was carried out in 20 μL volumes containing 0.2 mM dNTPs (Finnzymes, Finland), 0.2 μM primers (Generi Biotech, Czech Republic), 0.1 mg mL^-1^ bovine serum albumin (New England BioLabs, UK), 0.4 U of Phusion Hot Start II DNA Polymerase (Finnzymes, Finland) with the corresponding buffer, and template DNA (10–50 ng). The reaction conditions were as follows: 98°C for 3 min, 35 cycles of 98°C for 10 s, 60°C (*bphA*) or 51°C (*benA*) for 30 s, and 72°C for 30 s with final extension at 72°C for 10 min.

Resulting PCR products were purified using AMPure XP Beads (Agencourt, Beckman Coulter, USA) according to manufacturer’s instructions and pooled together prior to sequencing. Amplicons were unidirectionally sequenced from the forward primers using GS FLX+ system with Titanium reagents (Roche, Germany). The data were processed by amplicon analysis of signal processing.

### Data Processing

A general workflow was used for FLX+ Titanium data processing as follows: (i) separate reads into samples by the exact match of barcode and primer sequence; (ii) trim off barcode and primer sequences; (iii) apply filtering/denoising algorithms, which are described further; (iv) trim the reads to the length of 400 bp while discard shorter sequences; (v) identify and eliminate chimeric sequences by UCHIME *de novo* (Edgar et al., 2011) with default settings; and (vi) correct FS by FrameBot. Sequence data manipulations, such as dereplication, sorting, and database searches, were done in USEARCH v7.0 (Edgar, 2010) and mothur v1.31.0 (Schloss et al., 2009) software programs.

The filtering/denoising tools used in the step (iii) were as follows:

(a) AmpliconNoise: A modified pipeline of AmpliconNoise v1.29 (Quince et al., 2011) was followed except that the chimera check by Perseus was replaced by UCHIME *de novo*. The modification was performed by implementing a custom script CleanOpt.pl in the “filter” step (Gaspar and Thomas, 2013) instead of CleanMinMax.pl with truncation step disabled (option 2).
(b) MEE calculation: Implementation of MEE filtering by R. C. Edgar was used. The extraction of reads and sequence manipulation was carried out by a collection of USEARCH v7.0 FASTAQ commands (Edgar, 2010) with supplementary python scripts available at www.drive5.com. For this study, the MEE value was set in the range 0.5–2.0 by 0.5 steps. To keep the highest quality sequences, all discarded sequences identical to valid sequences were re-extracted and the abundance numbers were updated.
(c) Single linkage pre-clustering (SLP): Modified single linkage clustering exploiting abundance information of sequences was performed as reported by Huse et al. (2010), with the clustering step of 1% difference (*w* = 0.01). Pairwise distances were calculated by pairwise.seqs command in mothur software project (Schloss et al., 2009).

### FrameBot

Frameshift correcting tool FrameBot v1.0 (Wang et al., 2013) was run locally with glocal alignment settings, FS penalty set to -15 and identity threshold of 0.4. Target protein sequences were obtained via FunGene Repository (Fish et al., 2013) using pre-built *bphA1* and *benA* sub-databases with filter settings of Minimum Score 300 and Minimum Size 450 amino acids (February 2014). Downloaded protein sequences were trimmed with used primers to match experimental amplicon size, and dereplicated. Alternatively, a novel *de novo* method of FS detection was proposed (see Results). A script which performs FrameBot *de novo* is presented in the Supplementary Material to the study (Supplementary Script 1) or is available at https://github.com/strejcem/FBdenovo. The tool was also implemented into FunGene Pipeline available at http://fungene.cme.msu.edu/FunGenePipeline/.

### FS Detection in Processed Sequences

DNA sequences from all treatments were concatenated and dereplicated in mothur. Unique sequences were searched by locally run BLASTX (BLAST+ v2.29, Camacho et al. (2009)) against NCBI non-redundant protein (nr) database (downloaded in February 2014). A custom R (R Development Core Team, 2009) script was written which reads a names file and a BLAST output file, back-replicates counts to the original treatments, and exports results to a table showing total and unique numbers of sequences and numbers of sequences containing FS. In the case of mock community sample, the database was made of protein sequences known to be present. This allowed for detecting the majority of possible FS as well as calculating amino acid accuracy of obtained sequences; accuracy was defined as the number of identical amino acid residues of the experimental sequences to their respective references divided by their expected full length (133).

### Sequence Diversity Analysis

DNA sequence data sets of the same gene from both soils were combined and translated into proteins. Multiple sequence alignment was performed by Muscle (Edgar, 2004) with default settings. The alignments were manually inspected and sequences of non-specific products were eliminated along with sequences with STOP codons. Phylogenetic trees were constructed in MEGA6 software (Tamura et al., 2013) by Neighbor-Joining method with a *p*-distance model and pairwise deletion as gap/missing data treatment.

A conservation analysis was performed by calculating frequencies of amino acids and gap-treated Shannon entropy (*H*′; Zhang et al., 2008) for each position of the multiple sequence alignments as described by Iwai et al. (2010).

### Sequence Deposition

Pyrosequencing reads were deposited in NCBI Short Read Archive under study accession number SRP059438.

## Results

### Data Treatments Comparison

**FIGURE 1:**
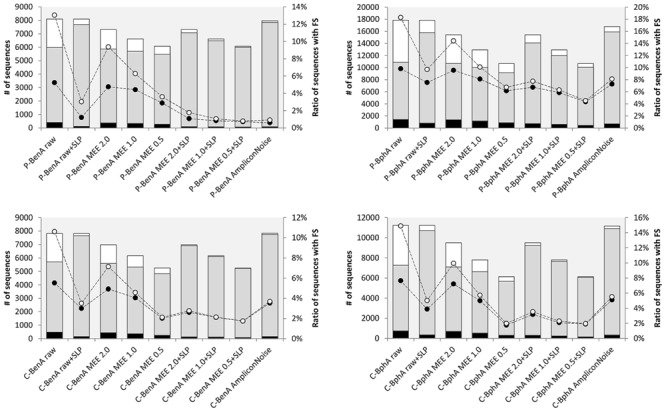
**Data treatment comparison before frameshift (FS) correction.** *P-BenA* (**left**, upper graph): BenA from the pristine soil; *P-BphA* (**right**, upper graph): BphA from the pristine soil; *C-BenA* (**left**, lower graph): BenA from the contaminated soil; *C-BphA* (**right**, lower graph): BphA from the contaminated soil. The bars represent number of sequences (left axis): 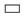 singletons; 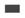 no singletons; 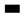 unique. The connected circles symbolize ratio of sequences with FS (right axis) to total sequences without singletons 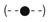 or ratio of sequences with FS to total sequences with singletons 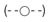.

Before correcting FS, the effects of AmpliconNoise, MEE filtering, and MEE filtering with SLP were evaluated using the following metrics: (i) total number of sequences; (ii) number of unique sequences; (iii) number of singletons; (iv) percent ratio of FS sequences including singletons; and (v) percent ratio of sequences excluding singletons. Ideally, the number of sequences after read processing should be approaching the original number of raw sequences and at the same time should contain minimum FS. By default, we considered singletons as very likely erroneous as demonstrated by the correlation between the number of singletons and FS sequences. The removal of singletons from raw data reduced the number of FS sequences on average by half; however, this effect became less evident with any further treatment, especially MEE with SLP or AmpliconNoise (**Figure 1**). For clarity, all numerical values reported throughout this manuscript are presented as averages from all four amplicon samples with standard deviations. Individual values are depicted in respective figures or tables.

Predictably, translated raw sequences suffered from the largest amount of FS sequences [14.2%, standard deviation *(SD*) = 2.8 percentage points (pp)] and singletons (31.9%, *SD* = 5.4pp). A notable decrease was achieved by either including SLP algorithm or applying AmpliconNoise. Both these treatments maintained very high number of total sequences (on average 11250, *SD* = 4037 and 10940, *SD* = 3634, respectively) while significantly decreasing the number of FS sequences (5.3%, *SD* = 2.6pp and 4.6%, *SD* = 2.6pp, respectively) and singletons (on average 5.9%, *SD* = 3.4pp and 2.6%, *SD* = 1.6pp, respectively).

MEE filtering in all cases resulted in a lower number of total sequences than the use of SLP or AmpliconNoise. Additionally, the number of sequences decreased with the strictness of MEE value, starting on average at 9809, *SD* = 3386 for MEE 2.0 and finishing at 7038, *SD* = 2133 for MEE 0.5. Combining MEE filtering with SLP, similarly to treating raw data with SLP, caused a decrease in the number of singletons (on average 4.0%, *SD* = 2.8pp for MEE 2.0 + SLP and 2.4%, *SD* = 2.1pp for MEE 0.5 + SLP) as well as unique sequences (for MEE 2.0 + SLP on average 323, *SD* = 256; for MEE 0.5 + SLP 189, *SD* = 158, **Figure 1**).

**FIGURE 2:**
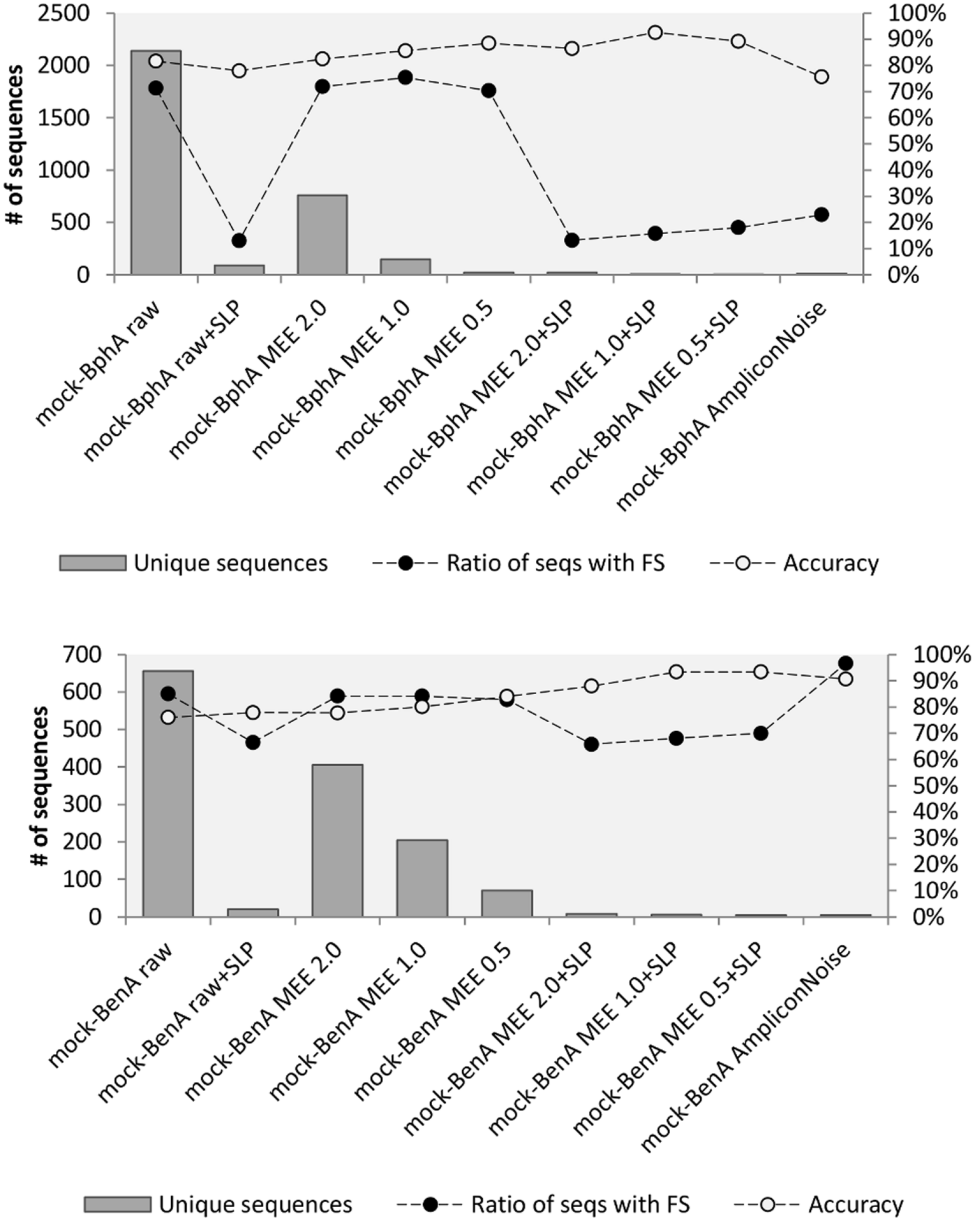
**Mock community analysis.** *Mock-BphA* **(top)**: BphA from the mock community sample; *Mock-BenA* **(bottom)**: BenA from the mock community. The 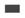 bars represent number of unique sequences (left axis). The connected circles symbolize ratio of sequences with FS (right axis) to total sequences 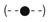 or accuracy of amino acid residues 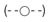.

The accuracy of all treatments was evaluated based on mock community data for both proteins. In case of BphA (**Figure 2**), the translation of raw sequences had the accuracy of 81.6% and 71.3% of FS sequences. The highest accuracy of 92.6% with 15.7% FS sequences was achieved by the application of MEE 1.0 in combination with SLP. Although comparable numbers of accuracy and ratio of FS were obtained by MEE 0.5 with SLP, this stricter criterion also resulted only in three unique sequences, while eliminating RHA1 strain BphA. Application of AmpliconNoise produced lower accuracy (0.756) than the bare translation of raw data (**Figure 2**). The analysis of BenA mock community showed again the same highest accuracy for MEE 0.5 and 1.0 in combination with SLP (93.4%) with the lowest FS ratio of 68.0% (**Figure 2**). AmpliconNoise produced similarly high accuracy of 90.7% but with the FS ratio of 96.6%.

### FS Correction

Frameshifts were detected in processed sets of sequences by BLASTX searches against NCBI nr protein database. In the case of mock community samples, the database was made of protein sequences known to be present, which allowed for detecting the majority of possible FS. Upon processing soil sample sequences through FrameBot, the number of sequences with FS reached zero.

### FrameBot *De novo*

**FIGURE 3:**
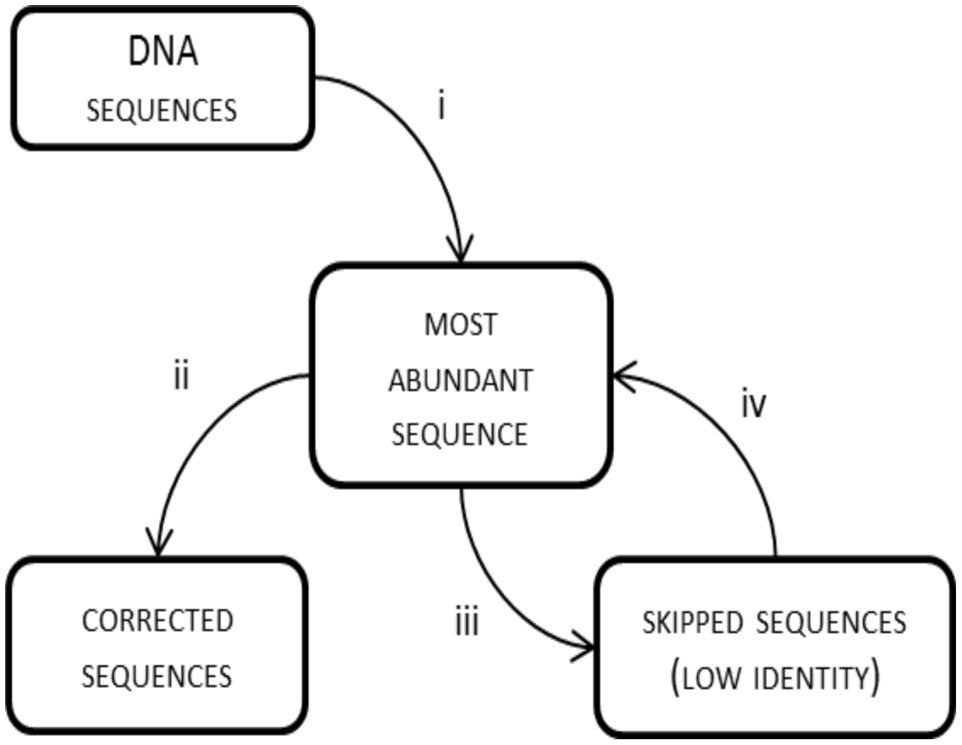
**FrameBot *de novo* scheme: (i) the most abundant experimental sequence is used as a reference for all sequences; (ii) FrameBot corrects sequences above identity cut off; (iii) sequences with low similarity to reference sequence are skipped; and (iv) the most abundant skipped sequence is used as a new reference sequence; if STOP codon is detected in a new reference, the sequence is put aside and will be checked with the next accepted reference; the cycle is repeated until there is no uncorrected sequence without STOP codon**.

By default, frame shifts are detected by translating experimental sequences in all reading frames, aligning them to protein references and searching for “frame breaks”. When reference sequences are closely related to the experimental ones, the entire process is effective. This was the case with the BenA sequences, where the average identity of experimental sequences to references was 83.1% and only 0–2% of reads (**Table 1**) were lost due to insufficient similarity to reference sequences. However, when the experimental reads are poorly related to references, the frame shift correction is inferior or true sequences are excluded from the analysis, which was apparent with BphA sequences from both soils. The average identity of BphA sequences to references was 42.5% and execution of FrameBot resulted in the loss of up to 12 and 44% of reads from BphA pristine and contaminated soil samples, respectively (**Table 1**). To overcome such issues, a *de novo* mode of FrameBot was proposed. The *de novo* mode is based on the assumption that erroneous sequences are derived from true sequences during the amplification or sequencing process, rendering abundant sequences to be more likely correct. The design of FrameBot *de novo* (**Figure 3**) can be separated into several steps: (i) experimental sequences are sorted by their abundance; (ii) the most abundant sequence is selected as the reference and is translated into a protein; (iii) FrameBot checks all the experimental sequences using the single reference, sequences below the identity and protein length cut-offs are not processed (default 0.4 and 100, respectively); (iv) the most abundant sequence from the unprocessed (i.e., discarded) sequences is selected and translated into a new reference; and (v) the procedure is repeated until there are no unprocessed sequences. In addition, when a new reference is selected, it is tested for a STOP codon presence. If positive, the next most abundant sequence is selected and tested instead.

**Table 1:** Frame shift corrections reported by FrameBot (FB; reference-based mode and *de novo* mode).

Data treatment	FB reference-based corrected (%)	FB *de novo* corrected (%)	FB reference-based sequences discarded (%)	FB *de novo* sequences discarded (%)
P_BenA 1.0 MEE + SLP	1.5	1.4	0.1	<0.1
P_BenA AmpliconNoise	1.0	1.0	0.3	0.3
C_BenA 1.0 MEE + SLP	9.6	8.8	1.1	0.5
C_BenA AmpliconNoise	9.5	9.7	2.1	1.3
P_BphA 1.0 MEE + SLP	2.7	1.9	10.1	3.4
P_BphA AmpliconNoise	4.7	4.5	12.3	4.7
C_BphA 1.0 MEE + SLP	0.6	2.4	41.2	0.4
C_BphA AmpliconNoise	0.8	6.0	43.6	0.6

*P, pristine soil; C, contaminated soil; MEE, maximum expected error filtration; SLP, single linkage pre-clustering.*

Applying FrameBot *de novo* on BenA sequences was comparable to reference-based FrameBot (**Table 1**). The aforementioned loss of up to 12 and 44% of BphA reads in pristine and contaminated soil samples, respectively, was significantly reduced by FrameBot *de novo*. At the same time, the number of reported FS correction was increased by up to five times compared to the reference-based FrameBot (**Table 1**).

Based on these results, the diversity of BphA and BenA sequences was determined as follows: after trimming off barcode and primer sequences and trimming the reads to the length of 400 bp, MEE filtering with value of 1.0 followed by SLP was applied and chimeric sequences with singletons were eliminated. The final step consisted of FS correction by reference-based FrameBot applied on BenA sequences and FrameBot *de novo* applied on BphA sequences (Supplementary Material Figure 1). The summary of original and final sequence quantity can be found in Supplementary Material Table 1.

### Diversity of BphA

**FIGURE 4:**
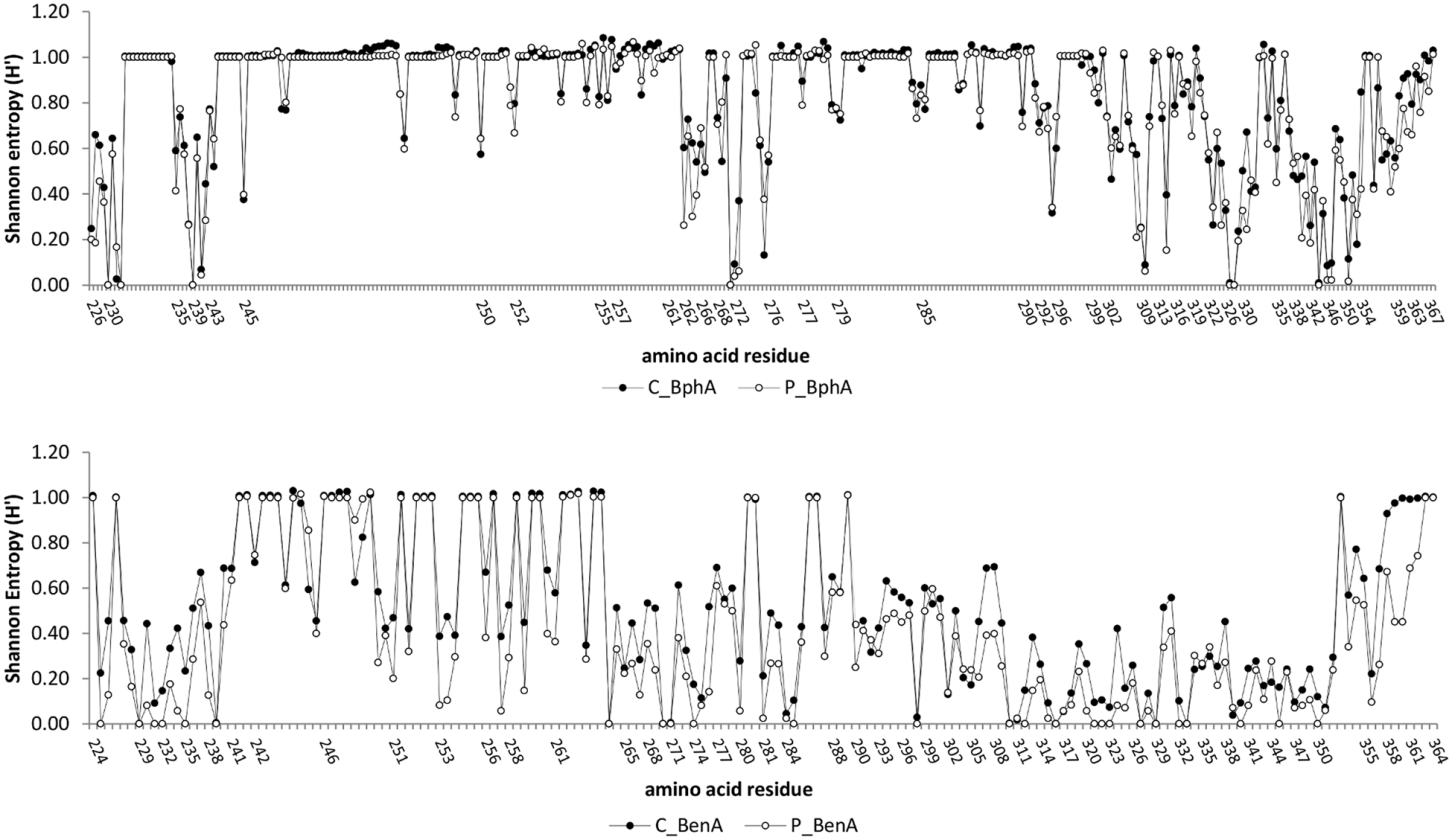
**Conservancy analysis of BphA (top) and BenA (bottom).** The numbers of amino acid residues correspond to those of *Burkholderia xenovorans* LB400 BphA and BenA. A conservation analysis was performed by calculating frequencies of amino acids and gap-treated Shannon entropy (*H*’) according to Zhang et al. (2008). High Shannon entropy is mostly a consequence of inserted gap in multiple sequence alignment over a majority of sequences.

Conservancy analysis indicated five highly conserved residues (frequency >99.9%) and additional two conserved residues (frequency >95%) shared in both contaminated and pristine soil BphA (**Figure 4**). Conserved amino acids were found in positions (LB400 numbering) Asp^230^, His^233^ and His^239^, Gly^271^, Phe^327^–Pro^328^, and Pro^344^. Very low entropy was also detected for contaminated soil BphA position 232 (mostly Tyr) and pristine soil BphA positions Gly^346^–Pro^347^ and Glu^351^. A phylogenetic tree of BphA was divided into seven phylogenetic groups (Supplementary Material Figure 2). Group I, II, and III formed a clade with known biphenyl dioxygenases from PCB-degrading bacteria. The first four most abundant sequences in the contaminated soil clustered in group I with BphA of Gram-negative bacteria and in group II with BphA from rhodococci. By far the most abundant BphA sequence in the pristine soil, located in phylogenetic group V, shared only ≤59% identity with previously described aromatic ring hydroxylating dioxygenases. The closest published matches to the sequences related to the most abundant pristine soil BphA sequence were phenoxybenzoate or 3-phenylpropionate/cinnamic acid dioxygenases with identities as low as 40%. A distinct cluster IV was related to 3-phenylpropionate dioxygenases, although no high identity matches to known sequences were found. The remaining clade consisted of phylogenetic groups VI and VII, and was most related to dioxygenases from uncultured bacteria. Overall, the phylogenetic tree of deduced amino acid sequences of BphA showed extensive diversity of these molecules in environmental samples with novel structures and possibly unknown substrate specificities (Supplementary Material Figure 2). Interestingly, there was not a single sequence that was common to both investigated contaminated and pristine environments.

### Diversity of BenA

The newly designed primer set *benA* 649f and *benA* 1100r (see Materials and Methods for details) flanks the C-terminal domain of the dioxygenase alpha subunit similarly to the primers that target *bphA* genes. *In silico* testing of the primer set using the *ProbeMatch Search* tool in FunGene (Fish et al., 2013) showed that the primers target genes encoding alpha subunits of: benzoate 1,2-dioxygenases, toluate 1,2-dioxygenases, 2-halobenzoate 1,2-dioxygenases, 3-phenylpropionate dioxygenases, and benzene 1,2-dioxygenases from both Gram-negative and Gram-positive bacteria.

The number of conserved sites in BenA was much higher than in BphA, with seven highly conserved and additional six conserved amino acid residues across all sequences (**Figure 4**). In addition, differences in sequences from contaminated and pristine soils were much higher than in BphA; BenA sequences were generally much less diverse in the pristine soil than the contaminated soil with 25 highly conserved and additional four conserved amino acid residues in the pristine soil. Six distinct clusters were defined in the constructed BenA phylogenetic tree (Supplementary Material Figure 3). Clusters number I, III, IV, and VI were associated with proteobacterial benzoate/benzene dioxygenases. BenA of known bacterial strains were located in four of the six clusters (Supplementary Material Figure 3). The most abundant sequences originating from the contaminated soil were located in cluster I and were closely related to *Pseudomonas* sp. GM74 (Supplementary Material Figure 3). Cluster II was associated predominantly with *Actinobacteria*, including *R. jostii* RHA1 BenA. More than 70% of BenA sequences from the pristine soil formed a third cluster with their closest relatives being BenA from *Pseudomonas* spp. (Supplementary Material Figure 3). Cluster V consisted solely of anthranilate dioxygenases and cluster VI contained sequences very dissimilar to those previously known (identities ∼40—50% to proteobacterial BenA).

## Discussion

Ecologically relevant functional genes are important markers for assessing the microbial functional potential of environmental communities. Analyses of protein coding genes are mostly performed after translation of nucleotide sequences into proteins as protein sequences more accurately reflect biological function and functional relatedness (Penton et al., 2013). As translation is often burdened with shifted reading frames, downstream processing of sequences can become very challenging. We compared different raw data processing steps that would minimize, or possibly eliminate the occurrence of FS sequences, and proposed a different mode of correcting FS sequences which we term FrameBot *de novo*.

Application of a common FrameBot resulted in the elimination of detected FS in all samples, but also resulted in elimination of a significant proportion of BphA sequences (**Table 1**). The requirement that the analyzed sequences need to be similar to those previously known is the most serious drawback of FrameBot. The herein proposed FrameBot *de novo* was able to overcome this drawback and was also able to dramatically reduce the detected FS in the case of BphA (**Table 1**). For BenA sequences, the performance of FrameBot *de novo* closely approached that of the reference-based mode (**Table 1**).

The performance of FrameBot *de novo* was compared with another *de novo* FS correcting tool – MetaGeneTack (Tang et al., 2013). Although the FS correction rates were comparable between the two tools, reads processed by FrameBot *de novo* resulted in higher positive-scoring matches in BLASTX search against the NR database. Interestingly, the number of unique subject sequences in the BLASTX also increased compared to MetaGeneTack corrected reads. This is most likely explained by the better ability of FrameBot *de novo* to locate the true position of the frame shift as was observed when mock community samples were analyzed. For example, when MetaGeneTack corrected a frame shift, it changed the reading frame upstream of the true frame shift position leaving several amino acid residues between corrected FS and the true FS position incorrectly translated.

By design, FrameBot *de novo* can introduce new FS to true sequences if a highly abundant sequence is erroneous, therefore, it is desirable to provide data of the highest quality prior to the execution of FrameBot. Significant error reduction can be achieved through several mechanisms as shown in **Figure 1**. To evaluate the effects of different data treatments on protein coding sequence data, FS were detected in processed data sets of sequences by BLASTX searches against protein database.

Read filtration based on quality scores (QS) provided by a sequencing technology is a convenient way of processing high-throughput sequencing data. It is fast and available on almost all sequencing platforms. MEE filtration is based on calculating the probable number of errors for each read from QS provided by the sequencing technology. Reads with higher number of expected errors than user defined value are discarded. MEE filtering is advantageous to commonly used standard QS averaging over reads where single poor qualities can be concealed by neighboring high qualities. In this work, we combined MEE filtering with SLP which joins closely related sequences that are most likely of the same origin but were created and multiplied via imperfect amplification or sequencing processes (Huse et al., 2010).

AmpliconNoise represents a sophisticated method for removing noise from 454 pyrosequenced amplicons. AmpliconNoise consists of two algorithms – PyroNoise and SeqNoise. PyroNoise attempts to correct pyrosequencing errors (mainly in homopolymers), while SeqNoise acts in a similar way as SLP – it clusters closely related sequences to reduce mainly PCR noise. Gaspar and Thomas (2013) inspected the ways in which AmpliconNoise reads flowgram data and wrote a revised version of the responsible Perl script. Using their CleanOpt.pl instead of default CleanMinMax.pl with the truncation step disabled led to an improved base calling and a production of longer sequences. AmpliconNoise does not filter sequences but rather alters them in an attempt to correct them. It can, however, inadvertently introduce errors in correct sequences as was shown previously (Gaspar and Thomas, 2013; Wang et al., 2013) and as we demonstrated in the analysis of BenA mock community sample (**Figure 2**). In the light of these observations, MEE filtering with SLP was chosen for the diversity analyses of BphA and BenA followed by FrameBot *de novo* for BphA and reference-based FrameBot for BenA diversity analyses.

Sequence analysis of dioxygenases demonstrated the extensive diversity of these proteins in soil with novel structures and possibly unknown substrate specificities. Some residues of BphA are directly responsible for substrate binding and other ones influence catalytic properties of the enzyme. Among these residues, so-called region III, corresponding to *B. xenovorans* LB400 BphA positions 335–341, is of an outstanding importance for substrate binding and specificity. More specifically, residues 335 and 336 impact on substrate binding and orienting (Barriault et al., 2004), while residues 338 or 341 impact on catalytic activity (Mohammadi and Sylvestre, 2005). We observed different patterns in the amino acid residues of this region in both pristine and contaminated soils. For instance, the most abundant BphA sequences from the contaminated soil were similar in the structure of the region III to the previously known BphA from PCB-degrading bacteria (Vézina et al., 2008). Furthermore, a sequence was detected from the contaminated soil which shared 95% identity to BphA from *B. xenovorans* LB400 but contained Ala^335^ and Met^336^ while amino acid residues 337–340 were identical to those in LB400. Previously it was shown that substituting Thr^335^ and Phe^336^ in LB400 for Ala^335^ and Met^336^ results in broadening of substrate specificity of the enzyme (Barriault and Sylvestre, 2004; Kumar et al., 2011). Region III amino acid patterns in sequences from the pristine soil were mostly quite different. This could indicate likely functional speciation of the enzymes based on the compounds commonly found in the surrounding environment – chlorobiphenyls in contaminated soil and possibly plant-derived natural compounds in the pristine soil. The roles of BphA in the environment have not yet been clearly elucidated, but there have been studies published which suggest the ecological role of BphA during the turnover of plant-derived compounds (Furukawa et al., 2004; Pham et al., 2012; Pham and Sylvestre, 2013).

The residue 339 in the contaminated soil proved to be the most conserved (**Figure 4**) among amino acid residues of the region III, which is in agreement with previously published data (Vézina et al., 2008; Iwai et al., 2010). However, in the pristine soil, the most conserved residue was that in the position 340. Residues corresponding to those of 233 and 239 in LB400 BphA (**Figure 4**) proved to be very conserved. The two histidines in these positions are crucial for the enzymatic function as they coordinate the mononuclear iron of the active center (Furusawa et al., 2004). Amino acids in region I, i.e., residues 236–238 (Mondello et al., 1997), were notably more different from those of previously known BphA sequences. For instance, PCB-degrading taxa have commonly Thr at the position 236 and Thr/Met at 237 (Kumamaru et al., 1998; Vézina et al., 2008). In both environmental datasets, we detected other amino acid residues to be more common (Supplementary Material Figure 4). Published data also indicated that the residue 321 was conserved among all BphA sequences, being either Gly or Ala (Vézina et al., 2008). Our data showed that, in addition to Gly and Ala, other residues were also common, including Val, Arg, Pro, Thr, Ser, Asn, Gln, Ile, or others (Supplementary Material Figure 4).

Much less information is currently available on BenA and related toluate, 2-halobenzoate, and anthranilate dioxygenases, which are classified as group II dioxygenases according to Nam et al. (2001). It has been previously shown that both benzoate and toluate dioxygenases catalyze the oxygenation of benzoates to the corresponding *cis*-1,2-dihydroxycyclohexadienes, including benzoates which are *meta*- and *ortho*-chlorinated or alkylated (Ge et al., 2002). The substrate specificity of the actual BenA is usually narrower than that of toluate dioxygenase, which is capable of transforming *para*-substituted benzoates unlike BenA (Ge and Eltis, 2003). Our data indicated that anthranilate dioxygenases, which commonly transform benzoate as well (Chang et al., 2003), formed a distinct cluster. Interestingly, sequences in all other clusters were homologous to benzoate and benzene dioxygenases (Supplementary Material Figure 3). Inspection of sequences listed in Functional Gene Repository (Fish et al., 2013) showed that many sequences fitting the model used for BenA were benzene dioxygenases. Even the primers designed for *benA* sequences hybridized *in silico* with some benzene dioxygenase sequences, suggesting a close sequential relatedness of those two enzymes.

Overall, the results of this study bring new insights into the ecological analysis of functional genetic markers. We demonstrated that any tested sequence treatment prior to translation is useful to reduce the number of errors. The proposal of FrameBot *de novo* will enable researchers to analyze functional ecological markers of disparate nature where the independence of previously described sequences is required. This tool can further improve gene isolation processes; especially in conjunction with a metagenome-complexity reducing methods like stable isotope probing (Uhlík et al., 2013) or sequence capture (Denonfoux et al., 2013). Using the model cases of aromatic dioxygenases we proved that FrameBot *de novo* can help fill the gaps in knowledge associated with diversity of these molecules by discovering novel clades with yet-to-be determined substrate specificities.

## Conflict of Interest Statement

The authors declare that the research was conducted in the absence of any commercial or financial relationships that could be construed as a potential conflict of interest.

## References

[B1] Barriault D. Lepine F. Mohammadi M. Milot S. Leberre N. Sylvestre M. (2004). Revisiting the regiospecificity of Burkholderia xenovorans LB400 biphenyl dioxygenase toward 2,2′-dichlorobiphenyl and 2,3,2′,3′-tetrachlorobiphenyl. *J. Biol. Chem.* 279 47489–47496. 10.1074/jbc.M406808200 15342625

[B2] Barriault D. Sylvestre M. (2004). Evolution of the biphenyl dioxygenase BphA from *Burkholderia xenovorans* LB400 by random mutagenesis of multiple sites in region III. *J. Biol. Chem.* 279 47480–47488. 10.1074/jbc.M406805200 15342624

[B3] Bopp L. H. (1986). Degradation of highly chlorinated PCBs by *Pseudomonas* strain LB400. *J. Indus. Microbiol.* 1 23–29. 10.1007/BF01569413

[B4] Camacho C. Coulouris G. Avagyan V. Ma N. Papadopoulos J. Bealer K. (2009). BLAST+: architecture and applications. *BMC Bioinform.* 10:421 10.1186/1471-2105-10-421 PMC2803857 20003500

[B5] Caporaso J. G. Kuczynski J. Stombaugh J. Bittinger K. Bushman F. D. Costello E. K. (2010). QIIME allows analysis of high-throughput community sequencing data. *Nat. Methods* 7 335–336. 10.1038/nmeth.f.303 20383131 PMC3156573

[B6] Chang H.-K. Mohseni P. Zylstra G. J. (2003). Characterization and regulation of the genes for a novel anthranilate 1,2-dioxygenase from *Burkholderia cepacia* DBO1. *J. Bacteriol.* 185 5871–5881. 10.1128/jb.185.19.5871-5881.2003 13129960 PMC193950

[B7] Cole J. R. Wang Q. Fish J. A. Chai B. Mcgarrell D. M. Sun Y. (2014). Ribosomal Database Project: data and tools for high throughput rRNA analysis. *Nucleic Acids Res.* 42 D633–D642. 10.1093/nar/gkt1244 24288368 PMC3965039

[B8] Denonfoux J. Parisot N. Dugat-Bony E. Biderre-Petit C. Boucher D. Morgavi D. P. (2013). Gene capture coupled to high-throughput sequencing as a strategy for targeted metagenome exploration. *DNA Res.* 20 185–196. 10.1093/dnares/dst001 23364577 PMC3628448

[B9] Edgar R. C. (2004). MUSCLE: multiple sequence alignment with high accuracy and high throughput. *Nucleic Acids Res.* 32 1792–1797. 10.1093/nar/gkh340 15034147 PMC390337

[B10] Edgar R. C. (2010). Search and clustering orders of magnitude faster than BLAST. *Bioinformatics* 26 2460–2461. 10.1093/bioinformatics/btq461 20709691

[B11] Edgar R. C. Haas B. J. Clemente J. C. Quince C. Knight R. (2011). UCHIME improves sensitivity and speed of chimera detection. *Bioinformatics* 27 2194–2200. 10.1093/bioinformatics/btr381 21700674 PMC3150044

[B12] Fish J. A. Chai B. Wang Q. Sun Y. Brown C. T. Tiedje J. M. (2013). FunGene: the Functional Gene Pipeline and Repository. *Front. Microbiol.* 4:291 10.3389/fmicb.2013.00291 PMC3787254 24101916

[B13] Furukawa K. Hayase N. Taira K. Tomizuka N. (1989). Molecular relationship of chromosomal genes encoding biphenyl/polychlorinated biphenyl catabolism: some soil bacteria possess a highly conserved bph operon. *J. Bacteriol.* 171 5467–5472. 2507526 10.1128/jb.171.10.5467-5472.1989 PMC210385

[B14] Furukawa K. Suenaga H. Goto M. (2004). Biphenyl dioxygenases: functional versatilities and directed evolution. *J. Bacteriol.* 186 5189–5196. 10.1128/JB.186.16.5189-5196.2004 15292119 PMC490896

[B15] Furusawa Y. Nagarajan V. Tanokura M. Masai E. Fukuda M. Senda T. (2004). Crystal structure of the terminal oxygenase component of biphenyl dioxygenase derived from *Rhodococcus* sp. *strain RHA*1. *J. Mol. Biol.* 342 1041–1052. 10.1016/j.jmb.2004.07.062 15342255

[B16] Gaspar J. M. Thomas W. K. (2013). Assessing the consequences of denoising marker-based metagenomic data. *PLoS ONE* 8:e60458 10.1371/journal.pone.0060458 PMC3607570 23536909

[B17] Ge Y. Eltis L. D. (2003). Characterization of hybrid toluate and benzoate dioxygenases. *J. Bacteriol.* 185 5333–5341. 10.1128/jb.185.18.5333-5341.2003 12949084 PMC193743

[B18] Ge Y. Vaillancourt F. H. Agar N. Y. R. Eltis L. D. (2002). Reactivity of toluate dioxygenase with substituted benzoates and dioxygen. *J. Bacteriol.* 184 4096–4103. 10.1128/jb.184.15.4096-4103.2002 12107126 PMC135208

[B19] Hurtubise Y. Barriault D. Powlowski J. Sylvestre M. (1995). Purification and characterization of the *Comamonas testosteroni* B-356 biphenyl dioxygenase components. *J. Bacteriol.* 177 6610–6618. 7592440 10.1128/jb.177.22.6610-6618.1995 PMC177515

[B20] Huse S. M. Welch D. M. Morrison H. G. Sogin M. L. (2010). Ironing out the wrinkles in the rare biosphere through improved OTU clustering. *Environ. Microbiol.* 12 1889–1898. 10.1111/j.1462-2920.2010.02193.x 20236171 PMC2909393

[B21] Iwai S. Chai B. Sul W. J. Cole J. R. Hashsham S. A. Tiedje J. M. (2010). Gene-targeted-metagenomics reveals extensive diversity of aromatic dioxygenase genes in the environment. *ISME J.* 4 279–285. 10.1038/ismej.2009.104 19776767 PMC2808446

[B22] Kumamaru T. Suenaga H. Mitsuoka M. Watanabe T. Furukawa K. (1998). Enhanced degradation of polychlorinated biphenyls by directed evolution of biphenyl dioxygenase. *Nat. Biotechnol.* 16 663–666. 10.1038/nbt0798-663 9661201

[B23] Kumar P. Gomez-Gil L. Mohammadi M. Sylvestre M. Eltis L. D. Bolin J. T. (2011). Anaerobic crystallization and initial X-ray diffraction data of biphenyl 2,3-dioxygenase from *Burkholderia xenovorans* LB400: addition of agarose improved the quality of the crystals. *Acta Crystallograph. Sect. F* 67 59–63. 10.1107/S1744309110043393 PMC3079973 21206025

[B24] Kurzawová V. Štursa P. Uhlík O. Norková K. Strohalm M. Lipov J. (2012). Plant-microorganism interactions in bioremediation of polychlorinated biphenyl-contaminated soil. *New Biotechnol.* 30 15–22. 10.1016/j.nbt.2012.06.004 22728721

[B25] Masai E. Yamada A. Healy J. M. Hatta T. Kimbara K. Fukuda M. (1995). Characterization of biphenyl catabolic genes of gram-positive polychlorinated biphenyl degrader *Rhodococcus* sp. *strain RHA*1. *Appl. Environ. Microbiol.* 61 2079–2085. 7793929 10.1128/aem.61.6.2079-2085.1995 PMC167480

[B26] Mohammadi M. Sylvestre M. (2005). Resolving the profile of metabolites generated during oxidation of dibenzofuran and chlorodibenzofurans by the biphenyl catabolic pathway enzymes. *Chem. Biol.* 12 835–846. 10.1016/j.chembiol.2005.05.017 16039530

[B27] Mondello F. J. Turcich M. P. Lobos J. H. Erickson B. D. (1997). Identification and modification of biphenyl dioxygenase sequences that determine the specificity of polychlorinated biphenyl degradation. *Appl. Environ. Microbiol.* 63 3096–3103. 9251195 10.1128/aem.63.8.3096-3103.1997 PMC168606

[B28] Nam J. W. Nojiri H. Yoshida T. Habe H. Yamane H. Omori T. (2001). New classification system for oxygenase components involved in ring-hydroxylating oxygenations. *Biosci. Biotechnol. Biochem.* 65 254–263. 10.1271/bbb.65.254 11302156

[B29] Pavlíková D. Macek T. Macková M. Pavlík M. (2007). Monitoring native vegetation on a dumpsite of PCB-contaminated soil. *Int. J. Phytoremediation* 9 71–78. 10.1080/15226510601139433 18246716

[B30] Penton C. R. Johnson T. A. Quensen J. F. Iwai S. Cole J. R. Tiedje J. M. (2013). Functional genes to assess nitrogen cycling and aromatic hydrocarbon degradation: primers and processing matter. *Front. Microbiol.* 4:279 10.3389/fmicb.2013.00279 PMC3775264 24062736

[B31] Pham T. T. M. Sylvestre M. (2013). Has the bacterial biphenyl catabolic pathway evolved primarily to degrade biphenyl? The diphenylmethane case. *J. Bacteriol.* 195 3563–3574. 10.1128/jb.00161-13 23749969 PMC3754558

[B32] Pham T. T. M. Tu Y. Sylvestre M. (2012). Remarkable ability of *Pandoraea pnomenusa* B356 biphenyl dioxygenase to metabolize simple flavonoids. *Appl. Environ. Microbiol.* 78 3560–3570. 10.1128/aem.00225-12 22427498 PMC3346365

[B33] Pieper D. H. Seeger M. (2008). Bacterial metabolism of polychlorinated biphenyls. *J. Mol. Microbiol. Biotechnol.* 15 121–138. 10.1159/000121325 18685266

[B34] Quince C. Lanzén A. Curtis T. P. Davenport R. J. Hall N. Head I. M. (2009). Accurate determination of microbial diversity from 454 pyrosequencing data. *Nat. Methods* 6 639–641. 10.1038/nmeth.1361 19668203

[B35] Quince C. Lanzen A. Davenport R. J. Turnbaugh P. J. (2011). Removing noise from pyrosequenced amplicons. *BMC Bioinformatics* 12:38 10.1186/1471-2105-12-38 PMC3045300 21276213

[B36] R Development Core Team (2009). *R: A Language and Environment for Statistical Computing*. Vienna: R Foundation for Statistical Computing.

[B37] Ryšlavá E. Krejčjk Z. Macek T. Nováková H. Demnerová K. Macková M. (2003). Study of PCB degradation in real contaminated soil. *Fresenius Environ. Bull.* 12 296–301.

[B38] Schloss P. D. Gevers D. Westcott S. L. (2011). Reducing the effects of PCR amplification and sequencing artifacts on 16S rRNA-based studies. *PLoS ONE* 6:e27310 10.1371/journal.pone.0027310 PMC3237409 22194782

[B39] Schloss P. D. Westcott S. L. Ryabin T. Hall J. R. Hartmann M. Hollister E. B. (2009). Introducing mothur: open-source, platform-independent, community-supported software for describing and comparing microbial communities. *Appl. Environ. Microbiol.* 75 7537–7541. 10.1128/AEM.01541-49 19801464 PMC2786419

[B40] Tamura K. Stecher G. Peterson D. Filipski A. Kumar S. (2013). MEGA6: Molecular Evolutionary Genetics Analysis version 6.0. *Mol. Biol. Evol.* 30 2725–2729. 10.1093/molbev/mst197 24132122 PMC3840312

[B41] Tang S. Antonov I. Borodovsky M. (2013). MetaGeneTack: ab initio detection of frameshifts in metagenomic sequences. *Bioinformatics* 29 114–116. 10.1093/bioinformatics/bts636 23129300 PMC3530910

[B42] Uhlík O. Ječná K. Macková M. Vlček C. Hroudová M. Demnerová K. (2009). Biphenyl-metabolizing bacteria in the rhizosphere of horseradish and bulk soil contaminated by polychlorinated biphenyls as revealed by stable isotope probing. *Appl. Environ. Microbiol.* 75 6471–6477. 10.1128/AEM.00466-09 19700551 PMC2765145

[B43] Uhlík O. Leewis M. C. Strejček M. Musilová L. Macková M. Leigh M. B. (2013). Stable isotope probing in the metagenomics era: A bridge towards improved bioremediation. *Biotechnol. Adv.* 31 154–165. 10.1016/j.biotechadv.2012.09.003 23022353 PMC3578049

[B44] Uhlík O. Wald J. Strejček M. Musilová L. Rídl J. Hroudová M. (2012). Identification of bacteria utilizing biphenyl, benzoate, and naphthalene in long-term contaminated soil. *PLoS ONE* 7:e40653 10.1371/journal.pone.0040653 PMC3396604 22808223

[B45] Vézina J. Barriault D. Sylvestre M. (2008). Diversity of the C-terminal portion of the biphenyl dioxygenase large subunit. *J. Mol. Microbiol. Biotechnol.* 15 139–151. 10.1159/000121326 18685267

[B46] Wang Q. Quensen J. F. Fish J. A. Kwon Lee T. Sun Y. Tiedje J. M. (2013). Ecological patterns of nifH genes in four terrestrial climatic zones explored with targeted metagenomics using FrameBot, a new informatics tool. *mBio* 4:e592-13. 10.1128/mBio.00592-13 PMC3781835 24045641

[B47] Weisman D. Yasuda M. Bowen J. L. (2013). FunFrame: functional gene ecological analysis pipeline. *Bioinformatics* 29 1212–1214. 10.1093/bioinformatics/btt123 23511542

[B48] Zhang S. W. Zhang Y. L. Pan Q. Cheng Y. M. Chou K. C. (2008). Estimating residue evolutionary conservation by introducing von Neumann entropy and a novel gap-treating approach. *Amino Acids* 35 495–501. 10.1007/s00726-007-0586-0 17710364 PMC7088136

[B49] Zhang Y. Sun Y. N. (2011). HMM-FRAME: accurate protein domain classification for metagenomic sequences containing frameshift errors. *BMC Bioinformatics* 12:10 10.1186/1471-2105-12-98 PMC3115854 21609463

