# Supplementary material for: Hunting Down Frame Shifts: Ecological Analysis of Diverse Functional Gene Sequences
Source: Front Microbiol. 2015 Nov 24;6:1267. doi: 10.3389/fmicb.2015.01267 (PMC4656815; doi:10.3389/fmicb.2015.01267)
Supplement: Supplementary file 2 [file Supplementary_Material.PDF]

Hunting down frame shifts: Ecological analysis of diverse functional gene sequences

Michal Strejcek, Qiong Wang, Jakub Ridl, Ondrej Uhlik

SUPPLEMENTARY MATERIAL

SM Figure 1. Overview of the final pipeline used in this study.

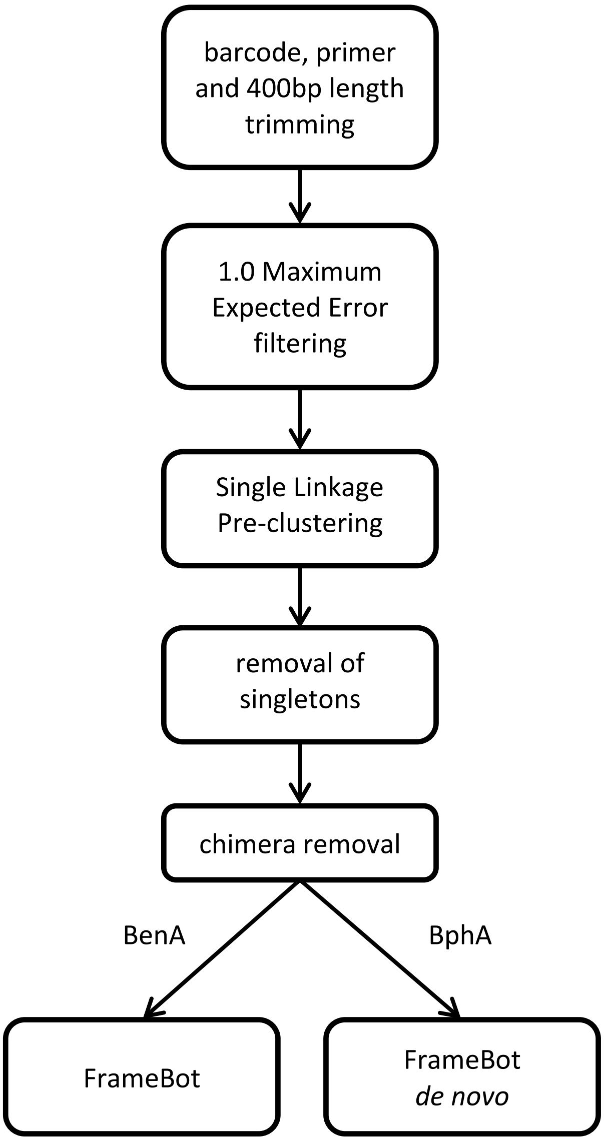

SM Figure 2. Phylogenetic tree of BphA.

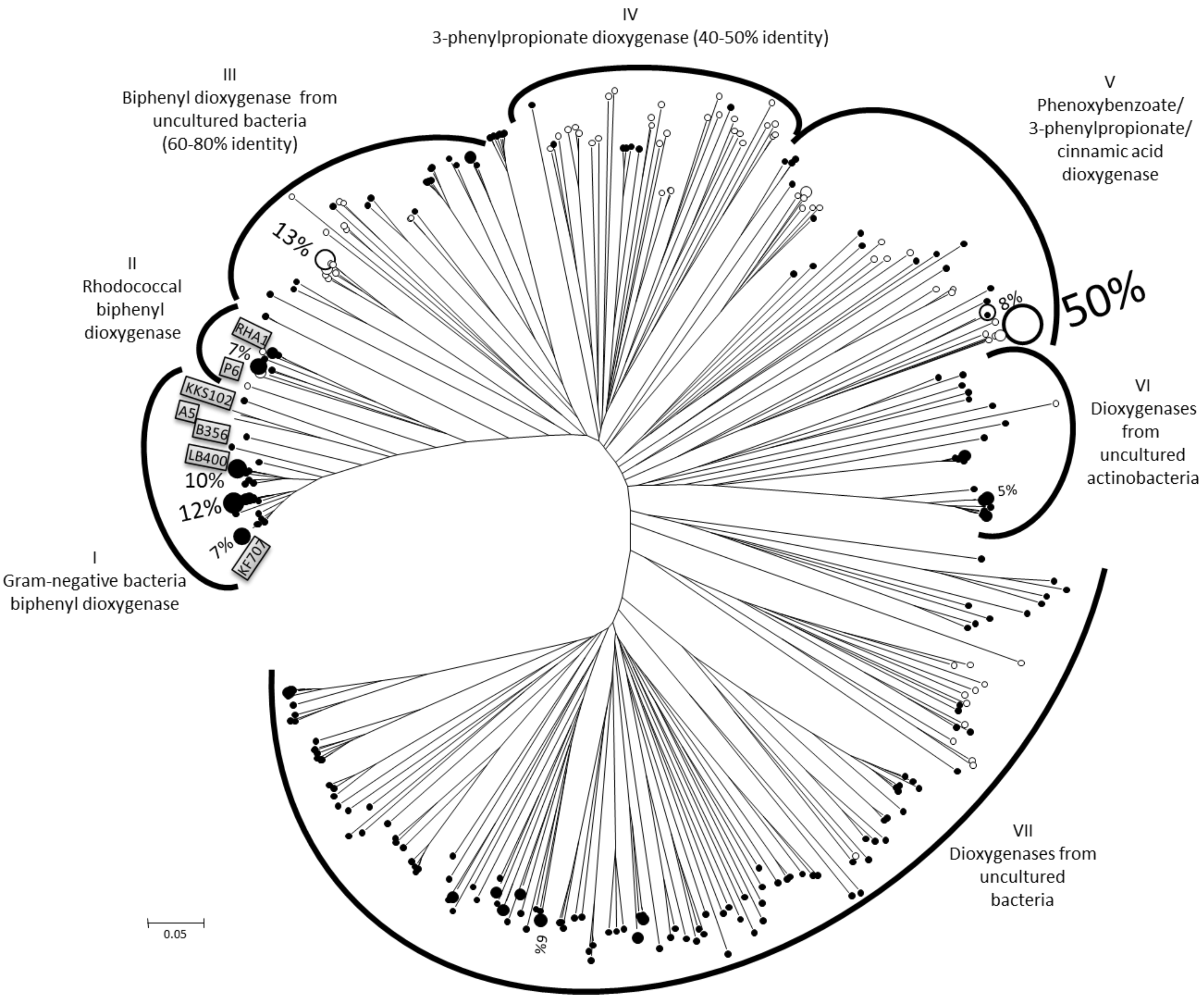

Symbols: ● BphA from contaminated soil, ○ BphA from pristine soil. Sequences >1% in abundance are shown as greater points, sequences >5% are labeled with particular percentage. The identity in brackets means sequence identity closest to entries in nr NCBI database. BphA sequences from model PCB-degrading bacteria are shown. Depicted BphA of strains: RHA1 – *Rhodococcus jostii* RHA1; P6 – *Rhodococcus globerulus* P6; KKS102 – *Acidovorax* sp. KKS102; A5 – *Cupriavidus oxalaticus* A5; B356 – *Pandoraea pnomenusa* B-356; LB400 – *Burkholderia xenovorans* LB400; KF707 – *Pseudomonas pseudoalcaligenes* KF707

SM Figure 3. Phylogenetic tree of BenA.

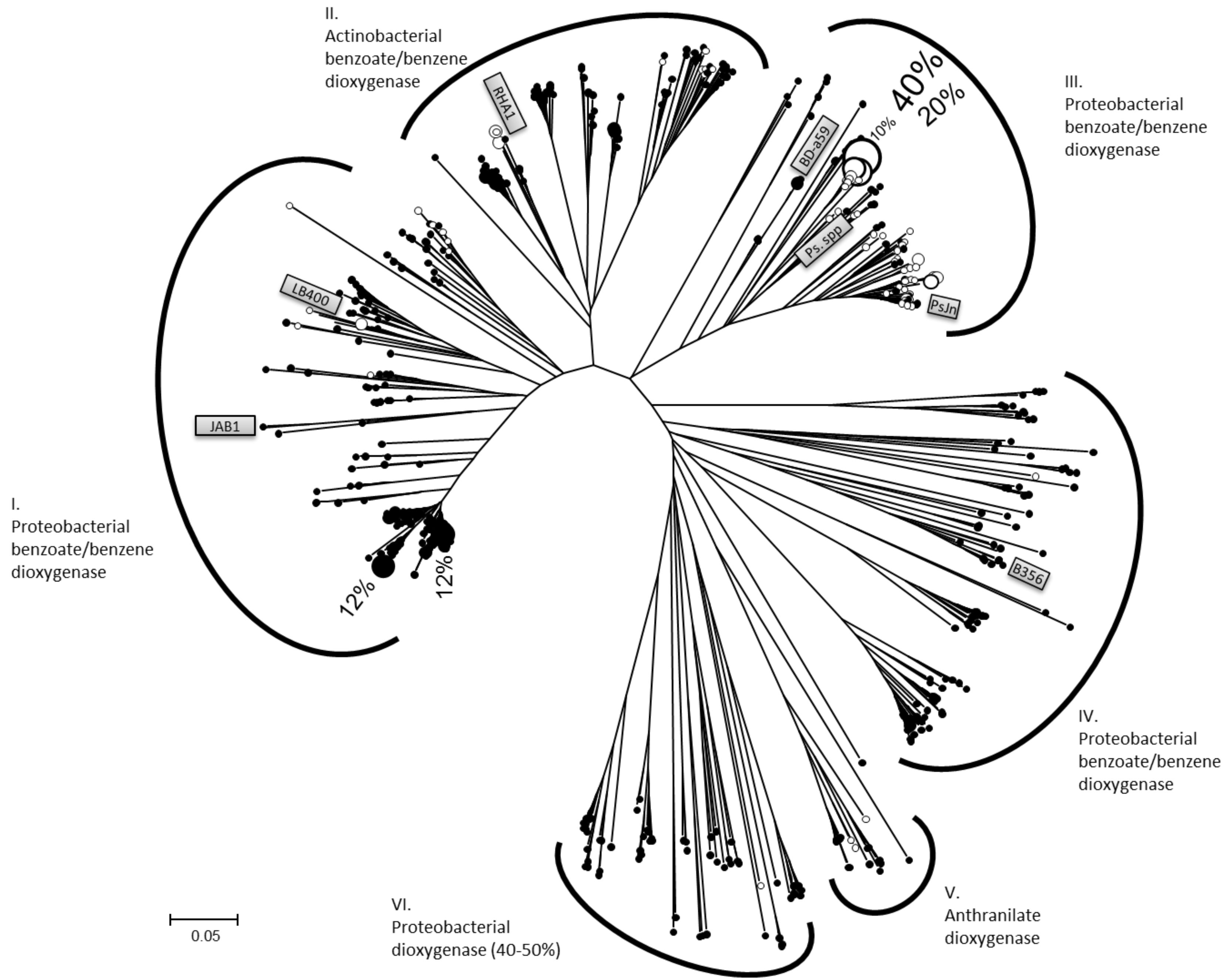

Symbols: ● BenA from contaminated soil, ○ BenA from pristine soil. Sequences >1% in abundance are shown as greater points, sequences >5% are labeled with particular percentage. BphA sequences from model PCB-degrading bacteria are shown. The identity in brackets means sequence identity closest to entries in NR NCBI database. Depicted BenA of strains: JAB1 – *Pseudomonas alcaliphila* JAB1; LB400 – *Burkholderia xenovorans* LB400; RHA1 – *Rhodococcus jostii* RHA1; BD-a59 – *Pseudoxanthomonas spadix* BD-a59; Ps. spp – MULTISPECIES (*Ps. brassicacearum* subsp. *brassicacearum* NFM421; *Ps. brassicacearum* 51MFCVI2.1; *Ps. fluorescens* Q8r1-96; *Ps. brassicacearum* PP1_210F; *Ps. fluorescens* Q8r1-96); PsJn – *Burkholderia phytofirmans* PsJN; B356 – *Pandoraea pnomenusa* B-356

SM Figure 4. Sequence logos of BphA multiple sequence alignment (LB400 numbering) for pristine soil (upper) and contaminated soil (lower) samples. Created with WebLogo (Crooks GE, Hon G, Chandonia JM, Brenner SE. WebLogo: A sequence logo generator, Genome Research, 14:1188-1190, (2004)).

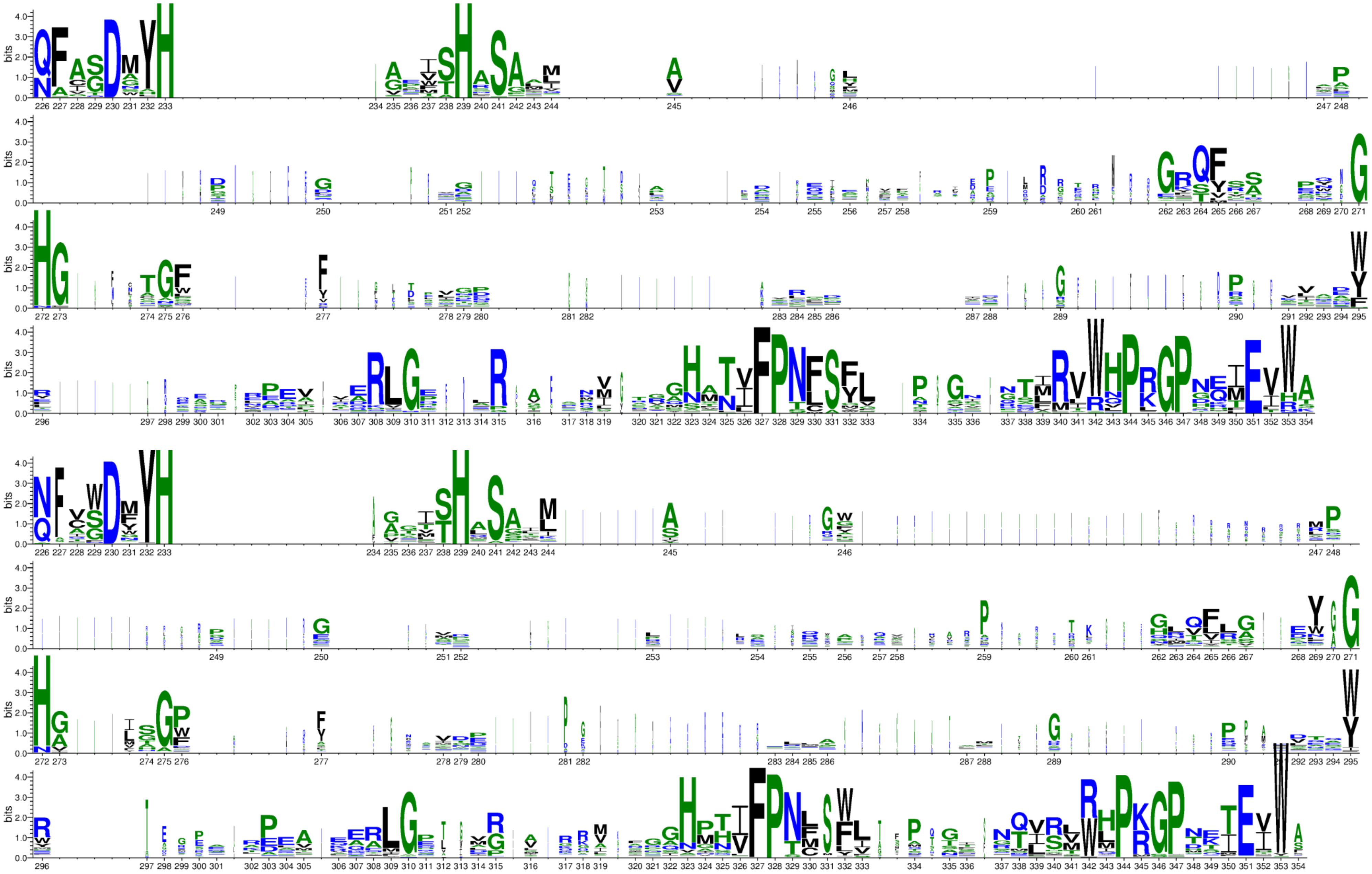

SM Table 1. Summary of the sequence data. P-*benA*: *benA* from the pristine soil; *P-bphA*: *bphA* from the pristine soil; C-*benA*: *benA* from the contaminated soil; C-*bphA*: *bphA* from the contaminated soil. "Final" means data reads after suggested workflow in Figure 3.

	unprocessed reads		after trimming to 400bp	final (no singletons)
	# of reads	avg. length	# of reads	# of reads
P-*benA*	8973	416	8103	4680
P-*bphA*	21752	417	17847	11862
C-*benA*	8563	459	7820	5210
C-*bphA*	11780	483	11228	6639
